# Impact of doctor-patient communication on preoperative anxiety: Study at industrial township, Pimpri, Pune

**DOI:** 10.4103/0972-6748.57852

**Published:** 2009

**Authors:** Vandana B. Nikumb, Amitav Banerjee, Gurleen Kaur, Suprakash Chaudhury

**Affiliations:** Department of Community Medicine, D Y Patil, Medical College, Pune - 411 018, India; 1 Department of Psychiatry, RINPAS, Kanke, Ranchi-834006, India

**Keywords:** Preoperative anxiety, Doctor-patient communication, Surgical patients

## Abstract

**Background:**

Anxiety may not be recognized by physicians though they affect a large number of patients awaiting surgery as reported in some studies. Good doctor-patient communication may have an impact on preoperative anxiety.

**Aim:**

To find out the incidence of anxiety in patients awaiting surgery and its association with good doctor-patient communication.

**Materials and Methods:**

The study was undertaken in a medical college hospital situated in an industrial township, for the duration of two months. It was a cross-sectional study. The study included 79 patients admitted to various surgical wards of a teaching hospital. Data was collected on a pretested questionnaire, which included a set of questions on various aspects of doctor-patient communication. The level of anxiety was assessed using the Hospital Anxiety and Depression Scale (HADS). Statistical analysis was carried out using the WHO/CDC package EPI INFO 2002. Though preoperative anxiety was collected on an ordinal scale, later during analysis, it was collapsed to give a categorical scale. Aspects of doctor-patient communication associated with preoperative anxiety were explored by Chi square tests.

**Results:**

Out of the total 79 patients, 26.5% reported definite anxiety levels. Good doctor-patient communication was found to be inversely associated with anxiety levels in the preoperative period.

**Conclusions:**

Preoperative anxiety is a common phenomenon among indoor surgical patients. A lot can be done to alleviate this anxiety by improving doctor-patient communication.

Anxiety is a complicating comorbid diagnosis in many patients with medical illnesses (Cukor *et al*., 2008). Persons who undergo surgical procedures are under strong preoperative distress (Chaudhury *et al*., 2006). It has been described that the incidence of preoperative anxiety varies from 11 to 80% in adults (Maranets and Kain, 1999). Preoperative anxiety and depression can also cause reactions that result in an increase in the intraoperative consumption of anesthetics and in a greater postoperative demand for analgesics (Caumo *et al*., 2001). Besides, preoperative anxiety and depression seem to have a profound influence on the immune system and on the development of infections. For prevention of preoperative anxiety there is a need to identify the associated factors which can be modified. This will help in postoperative recovery and patient satisfaction. In view of this the present study was carried out firstly, to find out the incidence of anxiety in preoperative patients and secondly to identify its association with good doctor-patient communication.

## MATERIALS AND METHODS

The study was carried out in a medical college teaching hospital. The medical college teaching hospital is located in the industrial township of Pimpri, Pune. Patients admitted for surgery during a two-month period were the subjects of study. A cross-sectional study design was used. Ethical clearance was obtained from the institutional committee.

### Inclusion criteria

Patients who were undergoing surgery during this period and were willing to participate in the study, were included.

### Exclusion criteria

All unconscious patients, patients with psychiatric disorders or on psychoactive drugs were excluded.

### Consent

After the selection of patients, informed consent was obtained.

### Collection of data

Those who agreed to take part in the study were asked to fill up a pretested questionnaire, the day before the surgery prior to pre-anesthetic evaluation. The main content of the questionnaire contained detailed demographic, socioeconomic and health status of the patient as well as questions relating to anxiety. Doctor-patient communication was assessed by ascertaining the knowledge of the patient regarding the surgical procedure, satisfaction about the information received, response to queries by the patient and trust in the treating physician. The level of anxiety among the patients was assessed using hospital anxiety and depression (HAD) scale (Hicks and Jenkins, 1988). This includes seven questions, each with four possible answers. As originally described, the HAD scale had 14 questions, seven scoring anxiety and seven scoring depression. We omitted the questions relating to depression. This scale has high sensitivity and specificity (Goldberg 1985), and has been used successfully in assessing anxiety in medically compromised patients (Tang *et al*., 2008). Interpretation of anxiety using the HAD scale: Each answer to the multiple choice questions had 0 to 3 points. Therefore the possible range of score was from 0 to 21. The interpretation was done as follows: A score of ≤ 7 meant that anxiety was not present, score of 8-10 indicated doubtful presence of anxiety, while scores ≥ 11 proved that anxiety was definitely present.

### Statistical analysis

Statistical analysis was carried out using EPI INFO 2002.

## RESULTS

Out of 79 patients 50(63.2%) patients reported anxiety levels ≤7 on the HAD scale, eight (10.1%) reported between 8-10 and 21(26.5%) fell in the category of ≥11.

### Knowledge about surgical procedure and anxiety levels

Patients who were well informed about the surgical procedure in advance had significantly less preoperative anxiety than those unaware of the procedure [Table 1].

**Table 1:** Information about the procedure of the surgery and anxiety levels

Has the doctor informed you about whole procedure?	Anxiety levels			Total
	≤7 (n = 50)	8-10 (n = 8)	≥11 (n = 21)	
Yes	33 (75.0)	6 (13.6)	5 (11.3)	44
No	17 (48.5)	2 (5.7)	16 (45.7)	35
Total	50	8	21	79

Figures in parentheses are percentages; For the purpose of statistical analysis column 1 and 2 were combined; Chi square value: 10.09; Degrees of freedom: 1; *P* = 0.001

### Satisfaction about the information given and anxiety levels

Similarly, those who were better satisfied with the information given by the physician suffered from significantly less anxiety levels [Table 2].

**Table 2:** Satisfaction about the information given and anxiety levels

Are you satisfied about the information given?	Anxiety levels			Total
	≤7 (n = 50)	8-10 (n = 8)	≥11 (n = 21)	
Yes	49 (75.3)	6 (9.2)	10 (15.3)	65
No	1 (7.1)	2 (14.2)	11 (78.5)	14
Total	50	8	21	79

Figures in parentheses are percentages; For the purpose of statistical analysis column 1 and 2 were combined; Chi square value: 20.439; Degrees of freedom: 1 *P* < 0.00

### Association between queries answered by the doctor and anxiety levels

It will be seen from Table 3 that when the doctor answered the queries to the patient’s satisfaction, the anxiety was significantly less.

**Table 3:** Queries answered by the doctor and anxiety levels

Has the doctor answered all of your queries?	Anxiety levels			Total
	≤7 (n = 50)	8-10 (n = 8)	≥11 (n = 21)	
Yes	45 (76.2)	5 (8.4)	9 (15.2)	59
No	5 (25.0)	3 (15.0)	12 (60.0)	20
Total	50	8	21	79

Figures in parentheses are percentages; For the purpose of statistical analysis column 1 and 2 were combined; Chi square value: 13.117 Degrees of freedom: 1 *P* < 0.00

### Association between trust in the doctor and anxiety levels

Similarly, those patients whose physicians evinced trust suffered significantly less from preoperative anxiety [Table 4].

**Table 4:** Trust in the doctor and anxiety levels

Do you trust the doctor?	Anxiety levels			Total
	≤7 (n = 50)	8-10 (n = 8)	≥11 (n = 21)	
Yes	49 (70.0)	7 (10.0)	14 (20.0)	70
No	1 (11.1)	1 (11.1)	7 (77.7)	9
Total	50	8	21	79

Figures in parentheses are percentages; For the purpose of statistical analysis column 1 and 2 were combined; Fisher’s exact test; *P* = 0.001

## DISCUSSION

With the advancement of technology and expertise in the field of medicine we are now able to give answers to many unsolved questions of the past. In the midst of all these answers we are now facing hurdles of new questions. In this era of many new surgical interventions, where even space can’t bind man, just the thought of going through the unknown may provoke intense worry and anxiety. Preoperative anxiety may go unnoticed in an environment that stresses increased productivity. However, preoperative anxiety has been reported to be associated with poor psychosocial outcome after surgery (Chaudhury *et al*., 2006). Preoperative anxiety is also related to postoperative pain. In a study of 80 patients, aged 18 to 70 years undergoing laparoscopic cholecystectomy, postoperative pain perception intensity was primarily predicted by sex with an additional role of depression and anxiety (De Cosmo *et al*., 2008). Mitchinson *et al*., (2008) studied 605 patients undergoing surgery and concluded that patients should be screened preoperatively for pain and anxiety because these are strong predictors of a more difficult postoperative recovery. It has also been observed that patients with a high level of preoperative anxiety respond worse to analgesic medication than patients with a low level of preoperative anxiety. Therefore actions undertaken to reduce patients’ anxiety may reduce patients’ need of analgesic medications (Greszta & Siemińska, 2008).

Hospitalization, even in patients who are not faced with the prospect of surgery, is known to cause anxiety. One may, therefore, expect some degree of anxiety in patients attending for surgery. In industrial belts this anxiety is compounded by the stress of migration from a rural background to an urban environment in search of jobs and opportunities provided by industries.

Better doctor-patient communication can improve mutual understanding of such symptoms (Zastrow *et al*., 2008). The hospital anxiety and depression scale (HADS) identifies patients with psychological distress who may benefit from early counseling (Awsare *et al*., 2008). The present study indicates that better doctor-patient communication which involves information sharing about the surgical procedure, patient satisfaction, attention to queries by the patient and trust in the physician was associated with lower anxiety levels. Similar findings have been reported by Herrera-Espineira *et al*., (2008), who reported that a greater anxiety level in patients was associated with greater dissatisfaction with information received. When a patient comes to a doctor for treatment he bonds with the doctor in such a way that he attributes almost divine powers to the physician. Therefore it becomes the duty of the doctor to help the patients allay his fears. This can be best done by developing a good rapport with the patient by listening patiently to his queries and answering them in an understandable manner. This would help the patient to trust the doctor and therefore feel satisfied. Hence good communication skills demonstrated by the doctor would reduce preoperative anxiety to a significant level.

### Limitation

The small sample size was an apparent limitation of the study.

## CONCLUSIONS

Preoperative anxiety is a common phenomenon in patients undergoing surgery. It can be reduced by better doctor-patient communication.
